# Towards the development of a comprehensive framework: Qualitative systematic survey of definitions of clinical research quality

**DOI:** 10.1371/journal.pone.0180635

**Published:** 2017-07-17

**Authors:** Belinda von Niederhäusern, Stefan Schandelmaier, Marie Mi Bonde, Nicole Brunner, Lars G. Hemkens, Marielle Rutquist, Neera Bhatnagar, Gordon H. Guyatt, Christiane Pauli-Magnus, Matthias Briel

**Affiliations:** 1 Clinical Trial Unit, Department of Clinical Research, University Hospital Basel, Basel, Switzerland; 2 Basel Institute for Clinical Epidemiology and Biostatistics, Department of Clinical Research, University Hospital Basel, Basel, Switzerland; 3 Department of Health Research Methods, Evidence, and Impact, McMaster University, Hamilton, Ontario, Canada; Johns Hopkins University Bloomberg School of Public Health, UNITED STATES

## Abstract

**Objective:**

To systematically survey existing definitions, concepts, and criteria of clinical research quality, both developed by stakeholder groups as well as in the medical literature. This study serves as a first step in the development of a comprehensive framework for the quality of clinical research.

**Study design and setting:**

We systematically and in duplicate searched definitions, concepts and criteria of clinical research quality on websites of stakeholders in clinical research until no further insights emerged and in MEDLINE up to February 2015. Stakeholders included governmental bodies, regulatory agencies, the pharmaceutical industry, academic and commercial contract research organizations, initiatives, research ethics committees, patient organizations and funding agencies from 13 countries. Data synthesis involved descriptive and qualitative analyses following the Framework Method on definitions, concepts, and criteria of clinical research quality. Descriptive codes were applied and grouped into clusters to identify common and stakeholder-specific quality themes.

**Results:**

Stakeholder concepts on how to assure quality throughout study conduct or articles on quality assessment tools were common, generally with no *a priori* definition of the term quality itself. We identified a total of 20 explicit definitions of clinical research quality including varying quality dimensions and focusing on different stages in the clinical research process. Encountered quality dimensions include ethical conduct, patient safety/rights/priorities, internal validity, precision of results, generalizability or external validity, scientific and societal relevance, transparency and accessibility of information, research infrastructure and sustainability. None of the definitions appeared to be comprehensive either in terms of quality dimensions, research stages, or stakeholder perspectives.

**Conclusion:**

Clinical research quality is often discussed but rarely defined. A framework defining clinical research quality across stakeholders’ individual perspectives is desirable to facilitate discussion, assessment, and improvement of quality at all stages of clinical research.

## Introduction

Clinical research is necessary to advance our knowledge and practice of diagnosing and preventing diseases and treating patients. However, its complexity and the regulatory requirements have significantly increased over the last few years, requiring an ever-rising level of scientific, methodological, regulatory and organizational know-how [1]. Global clinical research involves billions of dollars and millions of people, yet it is often poorly planned, inefficient, or “not useful”, leading to considerable waste of private and public funding [1–8]. Low quality research may not only result in misleading findings [9], but may also compromise safety and rights of patients.

The regulatory international “ethical and scientific quality standard for designing, conducting, recording and reporting trials”–the Good Clinical Practice (GCP) guideline developed by the International Conference on Harmonisation (ICH) aims to ensure that safety and rights of participants are protected and that trial data are credible [10, 11]. The GCP guideline is a widely disseminated and applied standard for the broad concept of clinical research quality. However, its limitations include development as an agreement between industry and regulatory experts and its focus on data accuracy and extensive formal requirements has been criticized as an unsuitable standard for investigator-initiated clinical research [12, 13]. The GCP guidelines lack a broad stakeholder consensus and a sound evidence-base [14, 15].

In academic clinical research, “quality” often relates to design and implementation from the standpoint of scientific rigor. Over the last two decades a large number of quality assessment instruments and checklists have focused on specific aspects of quality in the context of specific types of research (e.g. the Cochrane Risk of Bias (RoB) tool for randomized trials [16], the tool for Quality Assessment of Diagnostic Studies (QUADAS-2) [17], or the Risk Of Bias In Non-randomized Studies tool (ROBINS-I) [18]). Other instruments have addressed the reporting of results from specific study types (e.g. CONsolidated Standards for Reporting Trials (CONSORT) [19], STrengthening the Reporting of OBservational studies in Epidemiology (STROBE) [20], or Preferred Reporting Items for Systematic Reviews and Meta-Analyses (PRISMA) [21]) and accordingly the reporting of protocols (e.g. Standard Protocol Items: Recommendations for Interventional Trials (SPIRIT) [22]). The Grading, Recommendation, Assessment, Development, and Evaluation (GRADE) initiative addresses risk of bias and, in addition, imprecision, inconsistency, and indirectness as domains to assess the overall quality of a “body of evidence” for the development of evidence-based clinical guidelines [23]. These instruments and checklists are useful means to address specific aspects of quality but do not consider the research process itself.

In other research fields, including higher education [24], legal sciences [25], or political sciences [26], the assessment of overall research quality has been described as complex, ambiguous, and a “major issue”. Increasingly, efforts have been directed towards the development of comprehensive quality frameworks [27]. Such broader approaches to quality assessment should consider the extent to which research meets the needs and expectations of stakeholders, and therefore depends on their perspective. However, the stakeholders in clinical research are numerous and their particular interests and priorities differ. Measurements of quality of clinical research may therefore be limited, or distorted, if prior consensus on a definition of quality has not been reached, and if the complexity of clinical research itself and the variety of stakeholders involved has not been taken into account. Avedis Donabedian, a pioneer in the assessment of the quality of care, declared in 1980: “What is missing (…) is a unifying theory of the definition and measurement of quality of care” (…) Before we attempt to assess the quality of care, either in general terms or in any particular site or situation, it is necessary to come to an agreement on what the elements that constitute it are” [28, 29].

This study aims to provide an overview of the existing definitions, concepts, and criteria of clinical research quality and to examine their variability by systematically synthesizing qualitative sources from the involved stakeholder groups and the medical literature. Clinical research in this context is defined as research conducted with patients to answer therapeutic, preventive, diagnostic, or prognostic questions or investigations of the mechanisms of human disease. We explicitly exclude research focusing on health care system processes, structures or policies (such as health services research or health technology assessments) and research with healthy volunteers. The findings of this study will inform the next step, i.e. the composition and structure of a comprehensive framework for clinical research quality as a common goal to increase value and reduce waste.

## Methods

We conducted two systematic searches for definitions, concepts, and criteria of clinical research quality (see Box 1 for definitions of terms). We searched (i) websites and any linked documents of stakeholders in clinical research, and (ii) the published medical literature.

**Box 1. Glossary of working definitions, in alphabetical order**

**Clinical Research**

Interventional and observational research addressing health care issues and involving human participants.

**Concept of quality**

An *implicit statement* on what clinical research quality means and comprises, e.g. which criteria are needed to ensure good quality research (often operational, e.g. “at our institution, the factors required to ensure quality are…“), or a discussion of one or multiple quality dimensions in the context of clinical research (e.g. internal validity, external validity, transparency, etc.).

**Definition of quality**

An *explicit statement* on what clinical research quality means and comprises, e.g. “quality of clinical research may be defined as the internal validity of study results and their applicability to patient treatment”, “quality of clinical research is commonly defined as…”, or “we define quality as…”. May include one or multiple quality dimensions.

**Quality criteria**

Aspects that are described as integral part(s) of quality, e.g. adherence to guidelines, use of standard operating procedures, etc.

**Quality dimension**

Overarching categories of quality criteria, e.g. internal validity, external validity, relevance, transparency, etc.

**Quality framework**

Theoretical foundation for a definition or concept of quality spanning multiple dimensions and study phases in a matrix structure; and serving the development of quality indicators for operationalization.

**Quality indicator**

An instrument to assess or measure an individual quality criterion, a group of quality criteria, or a quality dimension, i.e. the operationalization of quality criteria or dimensions (e.g. how to assess the adherence to guidelines).

**Quality theme**

A recurrent topic in the qualitative analysis of text material about quality definitions, concepts, or criteria extracted from stakeholder websites or articles published in the literature.

### Search of stakeholder websites

#### Stakeholder website selection

We searched stakeholder organizations (national ministry of health, regulatory body, pharmaceutical industry association, academic research organization, ethics committee, patients’ organization, funding agency, and initiative for clinical research) in 13 countries (Australia, Austria, Canada, France, Germany, Italy, Japan, Norway, Spain, Sweden, Switzerland, USA, UK) to provide perspectives from developed nations in different geographic regions. To identify at least one representative national stakeholder organization per stakeholder category in each of the 12 countries, we used personal contacts to one recognized expert in clinical research or public health per country. For the two contacts that did not respond (Australia, Norway), we identified the national organizations for all categories through a web search. We additionally searched for websites of inter- or supranational bodies involved in clinical research (e.g. ICH, WHO, Horizon2020, international associations) and the global 2013 Top10 pharmaceutical companies (IMSExecutive) and Contract Research Organizations (pharma-iq.com). We eventually identified publicly available websites of 155 organizations using the Google Search Engine (see S1 Table for the full list of screened organizations).

#### Eligibility criteria and search process

We systematically and in duplicate screened each website for a statement on a definition or concept of quality by the respective organization (e.g. “our trials are of high quality because they matter to patients”, or “quality means relevant, valid, and ethical trials”) using the keywords “quality” or “good” and “clinical research” or “clinical studies” or “clinical trials” or “research” in the website’s search function. If we did not find a statement on quality, we extended the search to related website content, e.g. “our policy”, “what we do”, “standards & quality assurance” etc., as well as organizational statements, guidelines, and reports. Within these documents we repeated the search for the above search terms using the respective search function. If no statements were found through the search function, the text was manually searched for paragraphs that described either a) the standards according to which the organization performed clinical research (i.e. ICH-GCP, Declaration of Helsinki, etc.), b) criteria according to which the organization assesses the quality of clinical research (e.g. evaluation criteria of funding programs), c) the processes used to assure the quality of clinical research within an organization (e.g. “quality assurance procedures”), or d) criteria which a “good study” should fulfil within the organization. We did not consider any statements that focused on animal research, quality of life, or quality of health care without providing any definition related to clinical research. For websites presented in languages other than English or German, text passages were translated by members of the investigative team (BvN, CPM, MMB, MR).

### Search of the literature (MEDLINE)

With the help of an experienced research librarian (NB) we designed a comprehensive search strategy using MeSH terms and text words (see S1 Text for full search strategy) and conducted a systematic literature search in MEDLINE using the Ovid interface from database inception to February 27, 2015. We did not impose any language restrictions.

#### Eligibility criteria and selection process

We included any article describing a definition, a concept, criteria, or a checklist, guideline, or measurement instrument of quality spanning more than one quality dimension of clinical research in general or within a specific clinical discipline. We excluded any articles not suggesting a definition, concept, or criteria of clinical research quality (e.g. exclusively discussing the implementation or validation of individual quality criteria or guidelines without providing any definition related to clinical research), systematic reviews applying an assessment tool of a specific aspect of quality (e.g. systematic reviews on the reporting quality of trials in a specific field applying CONSORT [19], or articles suggesting a measurement instrument/assessment tool of one specific aspect of quality (e.g. the Jadad Scale [30]). In addition, we excluded articles that focused on animal research, quality of life, or quality of health care without providing any definition related to clinical research.

Working in pairs, methodologically trained reviewers applied the pre-defined eligibility criteria independently after undergoing a calibration process. The reviewers first screened titles and abstracts. If titles and abstracts suggested an article meeting the above mentioned inclusion criteria or if eligibility remained unclear, we obtained corresponding full texts. Disagreements were resolved by discussion and consensus.

### Data extraction

We designed standardized extraction sheets suitable for qualitative data extraction (S2 Table) accompanied by an instruction manual. Before starting data extraction, the data extraction forms were piloted and teams of reviewers conducted calibration exercises to ensure consistency. We extracted text sections on the definition, concept, or criteria of quality from both literature and internet sources independently and in duplicate. Data synthesis of included articles involved categorization by overall topic, author, year of publication, article citation index (as retrieved in ISI Web of Science by 11 January 2016), and journal name. Internet sources were categorized by stakeholder group, country, and name of organization.

### Data analysis

We performed descriptive and qualitative explanatory analyses following the Framework Method [31] on definitions, concepts, and criteria of clinical research quality stratified by stakeholders and on evaluation criteria of funding agencies for clinical studies. The Framework Method belongs to a family of qualitative approaches termed thematic or content analysis, which identify commonalities and differences in qualitative data, and eventually seek to draw descriptive and/or explanatory conclusions clustered around themes. Its defining feature is the matrix output, i.e. rows (cases), columns (codes) providing a structure into which the researcher can systematically reduce the data in order to analyze it [32]. We therefore applied codes to excerpts of raw data and added or modified as new responses emerged. Codes were then grouped into clusters around similar and interrelated ideas to identify common and stakeholder-specific quality themes in an iterative process until consensus between the three investigators (BvN, MB, CPM) was reached. Themes were named after the most frequently recurring terms within the same clusters (e.g. generalizability, relevance, high quality data etc.) and were not created or imposed by the investigators.

## Results

### Definitions or concepts of clinical research quality in different stakeholder groups

We screened publicly available websites and linked documents of 155 stakeholders. Concepts of how to assure quality of clinical research or quality criteria were commonly reported among most stakeholder groups (66.4% (103/155); i.e. in 86.1% (31/36) of pharmaceutical companies or contract research organizations (CROs), 72% (18/25) of academic research organizations or initiatives, 63.6% (14/22) of international and governmental organizations, 61.9% (13/21) of regulatory agencies, 57.9% (11/19) of ethics committees, and 63.2% (12/19) of funding agencies, respectively), but this was relatively uncommon for patient organizations (31%; 4/13). However, only 12 of 155 (7.7%) institutions provided an explicit definition of the term ‘clinical research quality’ (pharmaceutical companies or CROs: 3/36; academic research organizations and initiatives: 3/25; international and governmental organizations: 3/22; regulatory agencies: 2/21; patient organizations: 1/13; ethics committees and funding agencies: 0/38) (S3 Table).

Qualitative analysis of the 12 definitions and the 103 quality concepts or criteria resulted in both common and stakeholder-specific quality themes often focusing on different stages of clinical research (planning/feasibility, conduct, dissemination; Table 1). Common quality themes amongst stakeholder groups included the adherence to all applicable national and international laws and regulations (e.g. ICH GCP), a scientific and methodologically rigorous approach allowing for an efficient and effective answer to the research question, credible and high quality data, the inclusion of trained study personnel, and the presence of Standard Operating Procedures (SOPs) and monitoring.

**Table 1:** Qualitative analysis of common and stakeholder-specific quality themes in the context of clinical research.

Stakeholder	Quality theme	Content / Explanation
**All stakeholders**	**Adherence to regulations & laws**	Trial performed, data generated, documented, recorded, reported in compliance with Declaration of Helsinki, ICH-GCP & all national and international applicable regulatory requirements Protection and respect for subject’s welfare, dignity and rights in accordance with Declaration of Helsinki
	**Scientific and methodological aspects of research**	Methodologically «sound» study and scientifically valid, effective & efficient answer to a scientific question Generation of credible and high quality data
	**Further common themes**	Qualified/trained personnel Presence of Standard Operating Procedures & adequate monitoring procedures
**Governmental bodies**	**Relevant, transparent, & ethical research**	Ability of a product, process, or service to satisfy stated or implied needs Public access to information and findings Impact on research community Integrity, preventing poor performance and misconduct
**Regulatory agencies**	**Adherence to guidelines**	Quality of evidence sufficient to support good decision making
**Academic research / Clinical Trial Units / Initiatives / Networks**	**Absence of bias, relevance &transparency**	Understanding of existing evidence, assumptions explicit and justified Particular focus on bias prevention, internal & external validity, methodological strength Advance knowledge, bear on policy issues, address needs of patients early The study should be compelling, useful, and relevant to stakeholders and decision makers The study should be objective, independent, and balanced Accurate reporting and transparency
**Pharmaceutical industry/ Contract Research Organizations**	**High quality data**	Fitness for purpose / use data Relevant to patients, HC professionals & society Publication of all scientifically and clinically relevant information
**Ethics committees / Institutional Review Boards**	**Risk/benefit ratio & subject protection**	Value enhancement of health or knowledge & benefit to community Favorable risk/benefit ratio Honesty, integrity, fair subject selection, free informed consent Acknowledgement of roles of others in research Responsible communication to the public
**Patient organizations**	**Patient involvement & applicability**	Feasible and practical trials, early patient involvement Patient-centeredness as to study procedures, inclusion/exclusion criteria and outcomes, impact on patient care Fair subject selection & Meaningful Informed Consent Access to quality information, during and after trial Access to treatment after trial Prevent risks and errors that truly matter to patient safety and the validity of the trial data
**Funding agencies**	**Feasibility, generalizability, & objectivity**	Overall feasibility, no duplication of research Important outcome to end user / potential clinical application Evidence on comparative effectiveness & cost Transparency / Reporting / Access to data Inter-/ multidisciplinarity No conflict of interest (financial/intellectual) Internationally competitive and reproducible capacity to attract resources

Stakeholder-specific emphasis on quality themes ranged from “high quality data” (pharmaceutical industry and CROs); “adherence to guidelines” (regulatory agencies); “patient involvement and applicability of research” (patient organizations); “absence of bias, relevance, and transparency” (academic research and/or initiatives); to “feasibility, generalizability, and objectivity of research” (funding agencies). The terminology used by the stakeholders to describe these themes (e.g. relevance, transparency, feasibility), was no less open to definition than the overarching concept of “quality” and as well depends on the perspective of the observer. In general, priorities within stakeholder groups were similar across different countries. However, for national funding agencies we found considerable variation in quality criteria that were particularly emphasized as relevant for funding decisions across countries (S4 Table).

### Definitions or concepts of clinical research quality in the medical literature

**Fig 1:**
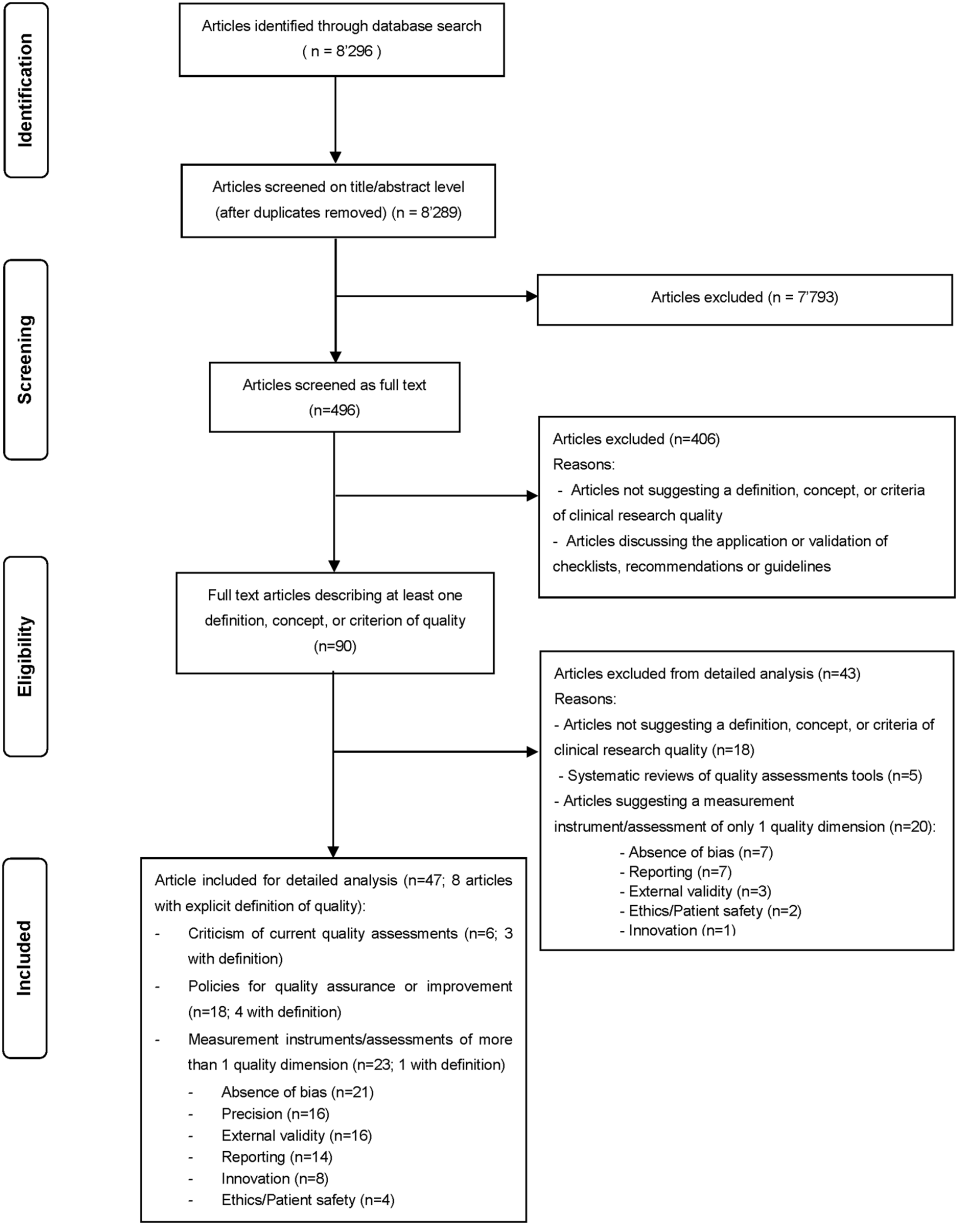
Article flow diagram.

Our systematic MEDLINE search yielded 8’289 titles and abstracts, of which we reviewed 90 articles in full text (Fig 1). We excluded 43 full text articles from detailed analysis, because they did not discuss a definition, concept, or criteria of quality (n = 18), they were systematic overviews/summaries of existing quality assessment checklists, instruments, or scores, with or without critical discussions of their validity and/or reliability (n = 5), or they discussed specific measurement instruments of a single dimension of quality (n = 20).

We included the remaining 47 articles for more detailed analysis (S2 Text). These provided concepts on how to assure or improve overall clinical research quality in specific contexts (e.g. at an academic institution, in a specific country, in a specific industry setting, or in a specific medical field; n = 18), or how to improve quality assessment (e.g. of RCTs, in radiology or hepatology research; n = 6). Measurement instruments or checklists that spanned more than one quality dimension were reported in 23 articles. A large proportion of these tools provided indicators on how to assess bias (n = 21). Almost half of them covered indicators on precision (n = 16), external validity (n = 16), or reporting quality (n = 14). Some tools additionally covered innovation aspects (n = 8) or ethical considerations (n = 4). None of the reviewed articles provided a definition or concept of clinical research quality spanning the encountered range of quality dimensions reflected by stakeholder perspectives such as ethical conduct, patient safety, patient values and preferences, absence of bias, precision, external validity, relevance, generalizability, transparency, infrastructure, and sustainability. Furthermore, we could not identify a definition or concept simultaneously covering several dimensions and differentiating between consecutive stages of research (e.g. study planning, conduct and dissemination), independent of a specific medical field or study setting.

Overall, we identified eight (8.9%) of 90 articles that provided an explicit definition of the term ‘clinical research quality’ (Table 2). The definitions therein span quality from methodological dimensions such as internal validity, external validity, or precision, and operational criteria including adherence to guidelines and applicable regulations (ethical conduct), to the effect of research at the societal level (relevance). None of the definitions appeared to be comprehensive either in terms of quality dimensions, research stages, or stakeholder perspectives. Five of the eight articles were cited less than 10 times in ISI Web of Science^™^ by 11.01.2016 (Table 2).

**Table 2:** Characteristics of articles providing an explicit definition of clinical research quality; by author (n = 8).

Author(s), Year	Title	Journal	Setting	Quality definition	Cit.^a^
Moher, Jadad et. al. (1996) [33]	Assessing the quality of randomized controlled trials. Current issues and future directions.	International Journal of Technology Assessessment in Health Care	RCTs	(…) Quality is a construct (concept) that can be defined in many ways, including the literary aspects for the report of a trial or its external validity, i.e. the degree to which it is possible to generalize trial results. Our focus on one important aspect of methodologic quality (hereafter simply "Quality"), internal validity, which we define as the "confidence that the trial design, conduct, analysis, and presentation has minimized or avoided biases in its Intervention comparisons." However, we recognize that this definition excludes other methodologic aspects of quality, for example, those concerned with the precision and reliability of measurements or estimation of compliance. (…)	244
Verhagen, de Vet et al. (2001) [34]	The art of quality assessment of RCTs included in systematic reviews	Journal of Clinical Epidemiology	Systematic review of RCTs	(…) Quality of RCTs has recently been defined as: “the likelihood of the trial design to generate unbiased results”. This definition covers only the dimension of internal validity. During the development of the “Delphi list” for quality assessment, the participants, all experts in the field of RCTs, failed to reach consensus on a specific definition, but did agree that the concept of quality should comprise more than internal validity alone. From this context we propose the following definition of quality: the likelihood of the trial design to generate unbiased results, that are sufficiently precise and allow application in clinical practice. (…)	125
Njie and Thomas (2001) [35]	Quality issues in clinical research and the implications on health policy (QICRHP)	Journal of Professional Nursing	General	(…) In this article, quality in clinical research is the process of developing and implementing guidelines to ensure the inclusion of all pertinent aspects of the research process, ensure accountability of research team members, adherence to protocol guidelines, and maintenance of study integrity and merit. (…)	1
Franck, Pendleton et al. (2004) [36]	Quality assurance for clinical research: challenges in implementing research governance in UK hospitals	International Journal of Health Care Quality Assurance Incorporating Leadership in Health Services	UK hospitals	(…) The essential elements of high quality research conduct derived from this body of literature are: research ethics (dignity, rights, safety, well-being of research participants); scientific quality, adherence to regulations (health and safety, medicines and devices); and information integrity (data protection, dissemination, financial and intellectual property). (…)	2
Switula (2006) [37]	The concept of quality in clinical research	Science & Engineering Ethics	General	(…) Quality in clinical research may be defined as compliance with requirements together with credibility and reliability of the data obtained. In the spirit of ISO, we may define quality in the clinical research process pictured above as the positive characteristics of the end product, that is the reliability and credibility of information collected during the clinical research process. Quality of research also means compliance of the whole trial process with pre-defined requirements. The customers of the clinical research define these requirements. (…)	3
Krestin (2008) [38]	Evaluating the Quality of Radiology Research: What Are the Rules of the Game?	Radiology	Radiology	(…)“I believe that research quality can be defined as the contribution of research to national and global social, economic, and scientific progress—that is, the effect of research at the societal level contribution of research to society.” (…)	1
Bhatt (2011) [39]	Quality of clinical trials: A moving target	Perspectives in Clinical Research	FDA	(…) Quality of clinical trials depends on data integrity and subject protection. (…)	8
Balshem, Helfand et al. (2011) [40]	GRADE guidelines: 3. Rating the quality of evidence.	Journal of Clinical Epidemiology	Quality of Evidence	(…)‘‘Quality” as used in GRADE means more than risk of bias and so may also be compromised by imprecision, inconsistency, indirectness of study results, and publication bias. In addition, several factors can increase our confidence in an estimate of effect. GRADE provides a systematic approach for considering and reporting each of these factors. (…)	690

^a^ Citations in Web of Science, last updated 11.01.2016 Abbreviations: FDA, US Food and Drug Administration; RCT, Randomized Controlled Trial; UK, United Kingdom

## Discussion

### Summary of findings

Our systematic review of stakeholder websites and the medical literature showed that quality of clinical research is frequently discussed, but rarely defined. Although stakeholder groups seem to agree on a basic concept of quality, their emphasis in the conceptualization of clinical research quality varies widely. The medical literature contains many articles discussing approaches to measurement or assessments of quality without prior definition of the term itself, and without reflecting the diversity of stakeholder needs, interests and expectations. A major proportion of these identified quality assessments aim to evaluate the “methodological rigor of randomized controlled trials”. The definition of “methodological rigor” in itself, however, varies substantially between the reported tools. Most authors suggested assessing methodological quality based on the presence or absence of measures to prevent bias. Others included dimensions such as external validity, reporting, or relevance of the study in question. We did not, however, identify a definition or concept including multiple dimensions or differentiating between consecutive stages of research across medical fields, or study settings. Although a comprehensive “definition” of quality may be difficult, a “concept” or “framework” of research quality, rather than a “definition”, could span all research stages and include more than an assessment focused on one aspect of quality. A more comprehensive approach to quality assessment, i.e. ranging from conceptualization to dissemination of a study as proposed by the authors of the Lancet series on “increasing value, reducing waste” [1, 4–7], rather than evaluation of the final published product, would assist in identification of errors that matter at earlier stages, and therefore support reduction of research “waste” more efficiently.

### Strengths and limitations

To our knowledge this is the first systematic survey addressing definitions and concepts of clinical research quality. Our systematic approach was suited to detect knowledge gaps, and to examine overlap and differences in perspectives of clinical research quality across stakeholder groups. Further strengths of this study include our consideration of websites and any linked documents from a large number of stakeholders in 13 different countries in addition to a Medline search. Methodologically trained investigators screened articles and websites in duplicate following a pre-specified instruction manual and undergoing a calibration process.

We acknowledge the following limitations: Although we consider our approach comprehensive, we searched only Medline as electronic database and relied on search terms in the title, abstract or other records. Articles in journals not indexed in Medline or providing some definition of research quality in the main text only might have been overlooked. However, Medline covers the most impactful journals and articles in current medical research and articles specifically focusing on quality of clinical research most likely mention this prominently. We may have missed definitions on websites despite screening these in duplicate. We would, however, expect stakeholder groups in clinical research to be transparent and proactive in defining such an important cornerstone of their activities, similarly to efforts in the field of clinical care quality. When coding our findings from the website search as well as from the Medline search we felt that we reached saturation, i.e. the last excerpts from websites or journal articles on aspects of clinical research quality did not bring new insights. The coding and qualitative analysis naturally involved subjective judgments of investigators, which we controlled by performing analyses in triplicate (BvN, CPM, MB) and comparing codes, findings, and interpretations until we reached consensus. Further, we acknowledge that our survey solely portrays perceptions on the quality of research in high income nations that may not necessarily overlap with those of low- or middle-income countries. With a growing percentage of clinical research being conducted in these geographies, a further study investigating quality perceptions of local stakeholders taking into consideration societal aspects and beliefs would be of importance. Finally, we did not conduct a detailed survey of experts or stakeholder groups—except for national funding agencies—nor did we conduct interviews with representatives of these groups to explore reasons for the paucity of explicit definitions.

### Comparison with other studies and implications

We are not aware of any other systematic survey on definitions, concepts, or criteria of overall clinical research quality. A similar approach has been taken by other authors to develop a framework for excellence, however with a distinct focus on translational cancer research [41]. In the field of health care quality, the focus of assessments has more and more shifted from process-based measurements towards the evaluation of patient outcomes and patient satisfaction [42–44]. Clinical research conducted in this context of “patient-centered” care would explicitly warrant the engagement and involvement of patients in setting priorities. However, patients (or their representatives) are only rarely considered when discussing the quality of research that might impact their care [45–47]. In our analysis, we also found patients to be surprisingly underrepresented. First, patient organizations were among the last in providing definitions or concepts of clinical research quality. Second, only six (14%) of a total of 43 quality measurement tools or assessments covered an item on patient safety and/or rights (Fig 1). Most efforts in quality assessments so far were taken to ensure compliance with guidelines, methodologically rigorous designs and valid study results. While this may ultimately serve the treatment of disease, we were expecting the clinical research enterprise to put unmet medical need and applicability to the patient population first. Compared with medical care, the clinical research machinery still seems to function with relatively low engagement of the end user (patients) of the product.

Furthermore, while we expected variations in the perception of quality across stakeholder groups, we were surprised how different and vaguely defined some of the concepts were. For example, an explicit definition of “high quality data” may be as dependent on the perspective of the observer as the definition of “high quality research”. It may be linked to concepts such as relevance of the data and absence of errors in the data or the way the data is collected. Similarly, the quality criteria used by funding agencies such as “impact”, “relevance”, or “feasibility” varied in their clarity and elaboration. Public funding agencies have a major role in terms of defining what and how research topics are investigated. Those who use these criteria to evaluate proposals are still left with subjective interpretation, while applicants may aim to provide the readers with these buzzwords with not much reflection on their meaning.

There remains considerable ambiguity in the use of current quality criteria across and within stakeholder groups. Unless carefully explained, these concepts can be easily misinterpreted by the stakeholders. Finding consensus on a common definition or concept of clinical research quality across national borders, stakeholder groups, and study types may therefore seem arduous; assessments of methodological quality do not, however, suffice. Existing quality guidelines such as ICH GCP have not been developed based on consensus across the full range of stakeholder groups, but only between regulatory experts and industry [15]. Existing quality assessment tools predominantly cover single aspects of quality, or particular research stages. Furthermore, there is a lack of approaches tailored to stakeholder requirements in assessing the quality of clinical research, e.g. from a patient’s perspective on how to choose a “good trial”, or from a funding agency’s perspective on how to assess the quality of studies before, during, and after the funding period. The authors of a follow-up study to the 2014 Lancet series reported that academic institutions in particular had paid only little attention to their recommendations on how to increase value in research. Practical guidance on how to implement these recommendations is so far lacking and urgently needed to increase value of academic research at all stages.

## Conclusions

This systematic survey serves as a first step of evidence summary to inform the development of a comprehensive framework of clinical research quality. It showed that definitions of clinical research quality are rarely provided and the existing definitions fall short of a theoretical or empirical framework across different study designs and stages and considering the variety of stakeholders involved. Based on our findings, a practically applicable framework needs to include the encountered quality dimensions such as ethical conduct, patient safety/rights/priorities, internal validity, precision of results, generalizability or external validity, scientific and societal relevance, transparency and accessibility of information, research infrastructure and sustainability) and consider different study stages such as planning, conduct, and dissemination. We plan to circulate framework drafts amongst stakeholder representatives of all eight groups until consensus on structure and content is reached, and to operationalize the framework through the development of instruments guiding stakeholder groups (e.g. academic institutions or funding agencies) in the comprehensive quality assessment of the full clinical research continuum.

## Supporting information

S1 Table List of all screened stakeholder organizations (n = 155). (DOCX)

S2 Table Data extraction forms. (DOCX)

S3 Table Quality definitions found through systematic internet search; by institution. Total number of institutions screened = 155.
(DOCX)

S4 Table Quality themes appearing in proposal evaluation criteria of funding agencies. (DOCX)

S5 Table PRISMA 2009 Checklist. (DOC)

S1 Text Literature search strategy. (DOCX)

S2 Text References of eligible articles. (DOCX)
