# Supplementary material for: Towards the development of a comprehensive framework: Qualitative systematic survey of definitions of clinical research quality
Source: PLoS One. 2017 Jul 17;12(7):e0180635. doi: 10.1371/journal.pone.0180635 (PMC5513422; doi:10.1371/journal.pone.0180635)
Supplement: S1 Table — (DOCX) [file pone.0180635.s001.docx]

**S1 Table. List of all screened stakeholder organizations (n=155)**

| **Stakeholder Category / Organization Name** | **Country/ Region** |
| --- | --- |
| **Governmental bodies / Jurisdiction** |  |
| World Health Organisation (WHO) | International |
| Organisation for Economic Co-operation and Development (OECD) | International |
| Department of Health | Australia |
| Bundesministerium für Gesundheit | Austria |
| Bundesinstitut für Qualität im Gesundheitswesen | Austria |
| Ministry of Health, Health Canada | Canada |
| Canadian Institute of Health Research | Canada |
| Ministère des solidarités, de la santé et de la famille | France |
| Ministère de l'enseignement supérieur et de la recherche (expert) | France |
| Bundesministerium für Gesundheit (BfG) | Germany |
| Bundesministerium für Bildung und Forschung | Germany |
| Ministero della Salute | Italy |
| Ministry of Health, Labour and Welfare (MHLW) | Japan |
| Ministry of Health and Care Services | Norway |
| Ministerio de Sanidad, Servicios Sociales e Igualdad | Spain |
| Socialdepartementet Sweden | Sweden |
| Bundesamt für Gesundheit (BAG) | Switzerland |
| National Health Service (NHS) (NIHR) | UK |
| Department of Health (DoH) | UK |
| National Health Service (NHS) | UK |
| National Institutes of Health (NIH) | USA |
| National Institutes of Health (NIH) (NINDS) | USA |
|  |  |
| **Regulatory Agencies/HTA Bodies** |  |
| International Conference on Harmonisation of Technical Requirements for Registration of Pharmaceuticals for Human Use (ICH) | International |
| Food and Drugs Administration (FDA) | USA |
| European Medicines Agency (EMA) | EU |
| Therapeutic Goods Administration (TGA) | Australia |
| [Bundesamt für Sicherheit im Gesundheitswesen / AGES Medizinmarktaufsicht](http://www.basg.gv.at/medizinprodukte) | Austria |
| Agence Nationale de sécurité du médicament et des produits de santé (ANSM) | France |
| Institut für Qualität und Wirtschaftlichkeit im Gesundheitswesen (IQWIG) | Germany |
| Bundesinstitut für Arzneimittel und Medizinprodukte (BfArM) | Germany |
| Zentralstelle der Länder für Gesundheitsschutz bei Arzneimitteln und Medizinprodukten (ZLG) | Germany |
| Gemeinsamer Bundesausschuss (GBA) | Germany |
| AgenziaFarmaco (AIFA) | Italy |
| Agenzia Nazional per i servizi sanitari (Agenas) | Italy |
| Pharmaceuticals and Medical Devices Agency (PMDA) | Japan |
| Legemiddelverket | Norway |
| Agencia Espanola del Medicamento y Productos Sanitarios (Agemed) | Spain |
| Läkemedelsverket Sweden | Sweden |
| Swiss Medical Board | Switzerland |
| Swissmedic | Switzerland |
| National Institute for Health and Care Excellence (NICE) | UK |
| Medicines and Healthcare Products Regulatory Agency (MHRA) | UK |
| General Medical Council | UK |
|  |  |
| **Pharmaceutical Associations / Companies / CROs** |  |
| **Associations** |  |
| International Federation of Pharmaceutical Manufacturers & Associations (IFPMA) | International |
| European Federation of Pharmaceutical Industries and Associations (EFPIA) | EU |
| Medicines Australia | Australia |
| Canada Pharma | Canada |
| Les entreprises du médicament (LEEM) | France |
| Verband Forschender Arzneimittelhersteller (VFA) | Germany |
| Farmaindustria | Italy |
| The Japan Pharmaceutical Manufacturers Association (JPMA) | Japan |
| Norwegian Association of Pharmaceutical Manufacturers (LMI) | Norway |
| Farmaindustria | Spain |
| Association of the British Pharmaceutical Industry (ABPI) | UK |
| Pharm. Research and Manufacturers of America (PhRMA) | USA |
| Läkemedelsindustriföreningen Sweden | Sweden |
| Interpharma | Switzerland |
| Scienceindustries | Switzerland |
| Verband der pharmazeutischen Industrie Österreichs (Pharmig) | Austria |
| Forum der forschenden pharmazeutischen Industrie in Österreich (FOPI) | Austria |
|  |  |
| **Top10 Pharmaceutical Manufacturers (2013)** |  |
| AstraZeneca | UK |
| Pfizer | USA |
| Pfizer | USA |
| Novartis | Switzerland |
| Merck | USA |
| Sanofi | France |
| Roche | Switzerland |
| GSK | UK |
| Eli Lilly | USA |
| Abbott | USA |
|  |  |
| **Top10 Clinical Research Organizations (2013)** |  |
| Target Health Inc. | USA |
| International Drug Development Institute (IDDI) | USA |
| Quintiles | USA |
| Parexcel | USA |
| PPD | USA |
| Covance |  |
| ICON | Other |
| Quotient Bioresearch | UK |
| Pharmanet (now InVentiv) | USA |
| Accovion | Germany |
|  |  |
| **Clinical Research Initiatives / Academic Clinical Research Organizations** |  |
| **Initiatives** |  |
| COCHRANE Collaboration | International |
| DEPLHI | International |
| All Trials | International |
| The San Francisco Declaration on Research Assessment (DORA) | International |
| RAND | International |
| Bill & Melinda Gates | International |
| STAR Child Health | International |
| PharmaTrain | EU |
| ECRIN | EU |
| Enpr-EMA European Network of Pediatric Research at the European Medicines Agency | EU |
| Instituto de Salud Carlos III | Spain |
| CIHR SPOR Network | Canada |
| Italian Cochrane Network | Italy |
| Center for Medicare & Medicaid Services | USA |
|  |  |
| **National Academic Research Organization Networks** |  |
| Swiss Clinical Trial Organisation (SCTO) | Switzerland |
| The Association of Canadian Academic Healthcare Organisations | Canada |
| Koordinierungszentrum für Klinische Studien (KKS Netzwerk) | Germany |
| AcademyHealth | USA |
|  |  |
| **National Clinical Research Associations** |  |
| Swiss Group for Clinical Cancer Research (SAKK) | Switzerland |
| Schweizerische Akademie der Medizinischen Wissenschaften (SAMW) | Switzerland |
| Clinical Research Association of Canada (expert) | Canada |
| Institut National de la santé et de la recherche médicale (INSERM): Reseau des CIC (expert) | France |
| Arbeitsgemeinschaft der Wissenschaftlichen Medizinischen Fachgemeinschaften (AWMF) (expert) | Germany |
| Deutsches Netzwerk Evidenz Basierte Medizin (EBM) (expert) | Germany |
| Japan Society of Clinical Trials and Research (expert) | Japan |
| UCSF Clinical Research HUB | USA |
|  |  |
| **Supranational and National Ethics Bodies** |  |
| World Medical Association (WMA), Declaration of Helsinki | International |
| Council for International Organizations of Medical Sciences(CIOMS), Ethical Guidelines for Biomedical Research Involving Human Subjects | International |
| European Group on Ethics in Science and New Technologies | EU |
| Australian Health Ethics Committee (NHMRC) | Australia |
| National Health and Medical Research Council, Australian Research Council Australian Vice-Chancellors’ Committee | Australia |
| Canadian Institutes of Health Research Standing Committee on Ethics | Canada |
| The National Consultative Ethics Committee for Health and Life Sciences | France |
| The German Ethics Council | Germany |
| Zentrale Ethikkommission bei der Bundesärztekammer | Germany |
| The National Bioethics Committee | Italy |
| The Council for Science and Technology, Subdivision on Research Planning & Evaluation, Bioethics & Biosafety Commission (MEXT) | Japan |
| The National Committees for Research Ethics (NEM) | Norway |
| The bioethics Committee of Spain (Comité de Bioética de España) | Spain |
| etikprövningsnämderna Sweden | Sweden |
| Swissethics | Switzerland |
| Secrétariat de la Commission cantonale (VD) d'éthique  de la recherche sur l'être humain | Switzerland |
| Central Office for Research Ethics Committees (COREC) | UK |
| Presidential Commission for the Study of Bioethical Issues | USA |
|  |  |
| **Supranational and National Patient Organizations** |  |
| European Patients' Academy on Therapeutic Innovation (EUPATI) | EU |
| European Patients Forum | EU |
| European AIDS Treatment Group | EU |
| Australian Patient Safety Organisation | Australia |
| Patients Canada | Canada |
| Collectif interassociatif sur la santé (CISS) | France |
| BundesArbeitsGemeinschaft der PatientInnenstellen und -Initiativen | Germany |
| Japan Patients Organisation | Japan |
| Foro espanol de pacientes | Spain |
| Positivrat | Switzerland |
| Patients Association | UK |
| National Breast Cancer Coalition and Nancy Roach, Colorectal Cancer Coalition (USA) | USA |
| National Patient Safety Foundation | USA |
|  |  |
| **Supranational and National Funding Agencies / Programs** |  |
| European Science Foundation (ESF) | EU |
| National Health and Medical Research Council | Australia |
| Canadian Institute for Health Research (CIHR) | Canada |
| Programme Hospitalier de Recherche Clinique | France |
| Deutsche Forschungsgemeinschaft (DFG) - Bundesministerium für Bildung und Forschung (BMBF) | Germany |
| Robert-Koch-Institut | Germany |
| Agenzia Italiana del Farmaco (AIFA) Fund | Italy |
| Ministry of Health (Research and Development Program) | Italy |
| Japan Society for the Promotion of Science (JSPS) | Japan |
| Japan Science and Technology Agency (JST) | Japan |
| The Research Council | Norway |
| Instituto de Salud Carlos III | Spain |
| Vetenskapsradet (Swedish Research Council) | Sweden |
| Schweizer Nationalfonds | Switzerland |
| Medical Research Council | UK |
| Patient Centered Outcome Research Institute (PCORI) | USA |
| NIH/NIH-The Common Fund | USA |
| Agency for Healthcare Research & Quality (AHRQ) (expert) | USA |
