# Supplementary material for: Towards the development of a comprehensive framework: Qualitative systematic survey of definitions of clinical research quality
Source: PLoS One. 2017 Jul 17;12(7):e0180635. doi: 10.1371/journal.pone.0180635 (PMC5513422; doi:10.1371/journal.pone.0180635)
Supplement: S2 Table — (DOCX) [file pone.0180635.s002.docx]

**S2 Table. Data extraction forms**

1. **Excel based extraction form for website search**

| **Organisation name** | **Location** | **Type of statement source** | **Type of quality statement** | **Text/Statement** | **Comments** | **Reference** | **Link** |
| --- | --- | --- | --- | --- | --- | --- | --- |
|  | International | Government Document | Definition |  |  |  |  |
|  | USA | Journal Article | Discussion |  |  |  |  |
|  | EU | Legal Rule or Regulation | Operationalisation |  |  |  |  |
|  | Australia | Magazine Article | Other |  |  |  |  |
|  | Canada | Personal Communication |  |  |  |  |  |
|  | France | Press Release |  |  |  |  |  |
|  | Germany | Report |  |  |  |  |  |
|  | Italy | Statute |  |  |  |  |  |
|  | Japan | Web Page |  |  |  |  |  |
|  | Norway | Guideline |  |  |  |  |  |
|  | UK | Other |  |  |  |  |  |
|  | Switzerland |  |  |  |  |  |  |
|  | Spain |  |  |  |  |  |  |
|  | Sweden |  |  |  |  |  |  |
|  | Austria |  |  |  |  |  |  |
|  | Other |  |  |  |  |  |  |

1. **Web-based full text extraction form for literature search**

| **Name** | **Type** | **Label** | **Options** |
| --- | --- | --- | --- |
| relevance | list | The article is relevant (according to inclusion/exclusion criteria, for details see "info") | yes |
|  |  |  | no, exclude |
| relevance | text | Why is article not relevant? Please comment. |  |
| article_type | list | The article's main focus is on | Definition of quality |
|  |  |  | Assurance/assessment of quality |
| data | list | Article results are based on | Expert consensus |
|  |  |  | Author opinion |
|  |  |  | Empirical data |
|  |  |  | Other |
| keytext | text | Copy key statement on quality definition from article |  |
| keyword | text | Quality Item/s (separated by ;) |  |
| dimension | list | To which quality dimension would you add the item/s? | Absence of bias |
|  |  |  | Precision |
|  |  |  | External validity |
|  |  |  | Innovation/Relevance |
|  |  |  | Reporting/Transparency |
|  |  |  | Education/Training |
|  |  |  | GCP/Patient safety |
|  |  |  | Other/New domain |
| scale | list | Was an existing quality scale/checklist/score used? | yes |
|  |  |  | no |
| namescale | text | Please provide the name of the scale/checklist/score used |  |
| dimchecklist | list | Which quality dimension/s does the checklist/scale assess? | Absence of bias |
|  |  |  | Precision |
|  |  |  | External validity |
|  |  |  | Innovation/Relevance |
|  |  |  | Reporting/Transparency |
|  |  |  | Education/Training |
|  |  |  | GCP/Patient safety |
|  |  |  | Other/New domain |
| indicators | list | Are additional indicators/metrics/measurement instruments or assessment methods of quality in clinical research mentioned? | yes |
|  |  |  | no |
| nameindicator | text | Please state what these indicators/metrics/instruments or assessments are: |  |
| addind | list | For which quality dimension are these indicators/metrics/assessments representative? | Absence of bias |
|  |  |  | Precision |
|  |  |  | External validity |
|  |  |  | Innovation/Relevance |
|  |  |  | Reporting/Transparency |
|  |  |  | Education/Training |
|  |  |  | GCP/Patient Safety |
|  |  |  | Other/new domain |
| comments | text | Comments |  |
