# Supplementary material for: Towards the development of a comprehensive framework: Qualitative systematic survey of definitions of clinical research quality
Source: PLoS One. 2017 Jul 17;12(7):e0180635. doi: 10.1371/journal.pone.0180635 (PMC5513422; doi:10.1371/journal.pone.0180635)
Supplement: S3 Table — Total number of institutions screened = 155. (DOCX) [file pone.0180635.s003.docx]

S3 Table. Quality definitions found through systematic internet search; by institution. Total number of institutions screened = 155.

| Organization | Geographic scope | Quality statement |
| --- | --- | --- |
| Governmental bodies / Jurisdiction | | |
| World Health Organisation (WHO) | International | (…) “Quality” is a measure of the ability of a product, process, or service to satisfy stated or implied needs. A high quality product readily meets those needs. In the context of a clinical trial, quality may apply to data (e.g., data are accurate and reliable) or processes (e.g., compliance with the study protocol and GCP; ensuring informed consent; adequate data handling and record-keeping, etc.). (See WHO GCP Principles 6: Protocol Compliance; 7: Informed Consent; 11: Records) (…) For all studies involving human subjects, even in the early stages (whether discovery or development), Good Clinical Practices are the correct quality and ethical standards. Tight national regulations ensure patient safety and methodological quality of clinical trials. (…) |
| Bundesamt für Gesundheit (BAG) | Switzerland | (…) **Art. 4** Scientific quality  The sponsor and the investigator of a clinical trial shall ensure scientific quality. In particular:  a.they shall define a research question based on the current state of scientific knowledge;  b.they shall use an appropriate scientific methodology; and  c.they shall ensure the availability of the resources required for the clinical trial and provide the necessary infrastructure (…) |
| Department of Health (DoH) | UK | (…)The key elements of a quality research culture are: • respect for participants’ dignity, rights, safety and wellbeing; • valuing the diversity within society; • personal and scientific integrity; • leadership; • honesty; • accountability; • openness; • clear and supportive management. (…) |
| Regulatory Agencies/HTA Bodies | | |
| Food and Drug Administration (FDA) | USA | (…) “Quality” is characterized by the ability to effectively and efficiently answer the intended question about the benefits and risks of a medical product (therapeutic or diagnostic) or procedure while ensuring protection of human subjects. (…)  Elements of a quality clinical study:   - Scientifically valid and ethically sound experimental design - Adequate protection of subjects rights, safety, and welfare - Qualified personnel - “Adequate” monitoring - Current, complete, and accurate data (…) |
| European Medicines Agency (EMA) | EU | (… ) Quality in this context is commonly defined as fitness for purpose. Clinical research is about generating information to support decision making while protecting the safety and rights of participating subjects. The quality of information generated should therefore be sufficient to support good decision making. (…) |
| Pharmaceutical Manufacturers & Contract Research Organizations | | |
| AstraZeneca | UK | (…) Quality in clinical research may be defined as… • Reliability and credibility of information providing an answer to a scientific question • Compliance of the trial process with defined requirements (Nach ISO 9000: A quality is a set of characteristics that a product or service must have to satisfy needs and expectations of the customer.  • Product of clinical research process: information.  • Customers of clinical research : Society, Research subjects, sponsors, regulatory authorities, hospitals/institutions, IECs (…) |
| Pfizer | USA | (…) Components for Quality: Clinical research quality is designed and embedded in the clinical trial processes and study protocol well in advance of enrollment of the first patient. Components of the quality process related to clinical trial sites include:  • Creating, implementing, and upholding standard operating procedures (SOPs) for trial execution  • A quality scientific and medical design of the protocol  • Clinical investigator and site pre-assessment and selection  • Regulatory agency and ethics committee approval  • Developing and providing appropriate informed consent (language, transparency of benefits and risks) and obtaining ethics committee approval of the informed consent process  • Investigator meetings and training  • Adequate recording and reporting of data  • Periodic monitoring  • Audits |
| Target Health Inc. | USA | (…) A “quality clinical trial” is one where 1) there is “absence of errors that matter” and 2) “are the data fit for use/purpose.” Errors “that matter” are those that have a 1) meaningful impact on patient safety and/or 2) Interpretation of trial results. (…) |
| Clinical Research Initiatives / Academic Clinical Research Organizations | | |
| COCHRANE Collaboration | International | (…) Quality : A vague notion of the methodological strength of a study, usually indicating the extent of bias prevention.(…) |
| DEPLHI | International | (…) Quality is a set of parameters in the design and conduct of a study that reflects the validity of the outcome, related to the external and internal validity and the statistical model used. (…) |
| Swiss Group for Clinical Cancer Research (SAKK) | Switzerland | (…) Quality is defined by several aspects in our organization. In general quality means the evaluation if we meet specific requirements in the development and conduct of our trials. These requirements are defined on different levels: a) The law (HRA): local applicable law to conduct clinical research b) In international guidelines (ICH GCP, GMP Annex 13, EU guidelines ect). c) International scientific trial specific standards d) Our internal requirements (e.g. internal requirements to conduct trials with high risks (phase I trials), which go further than what is specified e.g. in the law) (…) |
| Supranational and national patient organizations | | |
| National Breast Cancer Coalition and Nancy Roach, Colorectal Cancer Coalition (USA) | USA | (…)what “quality” means, i.e., what truly matters, to patients themselves:  • (…)“quality” and risk-based quality management requires patient-centred clinical trials that are scientifically valid and designed to robustly, efficiently answer questions of true import to patients, rather than questions that are simply of scientific interest but ultimately would have little impact on enhancing patient care.  • It requires trials that are designed to prevent risks and errors that truly matter to patient safety and the validity of the trial data.  • In addition, quality means patient-centred trials that appropriately incorporate patient preferences into study design and comprise “rational” design that minimises patient burden and maximises patient benefit.  And from the patients’ perspectives, “quality” also is defined by certain “don’ts”:  • quality trials are those that do not introduce invasive and/or repeated procedures, unnecessarily numerous study visits, and unnecessary costs for patients that are not required for answering the trial’s questions.  • They do not introduce unnecessarily restrictive inclusion and exclusion criteria that hamper accrual and may generate data that do not accurately reflect safety and efficacy for the larger patient population.  • And participation in such trials does not require unneeded delays in treatment initiation secondary to screening and trial arm assignment.  Coming full circle, quality trials provide uniformity in recruiting patients; are feasible and “practical” for both patients and their providers; include patient-centred, patient-friendly informed consents that truly inform patients; continually keep trial participants informed—whether the results are positive or negative; and move our body of knowledge forward and/or change practice. |
