# Supplementary material for: Towards the development of a comprehensive framework: Qualitative systematic survey of definitions of clinical research quality
Source: PLoS One. 2017 Jul 17;12(7):e0180635. doi: 10.1371/journal.pone.0180635 (PMC5513422; doi:10.1371/journal.pone.0180635)
Supplement: S4 Table — (DOCX) [file pone.0180635.s004.docx]

**S4 Table. Quality themes appearing in proposal evaluation criteria of funding agencies**

**A) Overview of quality criteria used by national funding agencies**

| Overarching quality themes | AU | AUS | CAN | CH | DE | UK | USA | NOR | Horizon 2020 |
| --- | --- | --- | --- | --- | --- | --- | --- | --- | --- |
| Quality of researcher, applicant, and/or team | x | x | x | x | x | x | x | x |  |
|  |  |  |  |  |  |  |  |  |  |
| Impact and/or significance of research project | x |  | x | x |  | x | x | x | x |
| Quality of scientific approach and/or methods | x |  | x | x |  | x | x | x |  |
| Research infrastructure & financial capacity at applying site |  |  | x |  | x | x | x | x | x |
| Consideration of diversity & equal opportunities of applicants |  | x |  |  | x |  |  | x |  |
| Ethical considerations of research project | x |  |  |  |  | x |  | x |  |
| Originality/innovation of research project |  |  | x | x |  |  | x |  |  |
| Feasibility of research project | x |  |  | x |  |  |  |  |  |
| Requested resources or funding adequate for proposed project |  |  |  |  | x | x |  |  |  |
| Quality of international / national cooperation arrangements |  |  |  |  | x |  |  | x |  |
| Dissemination & communication of results |  |  |  |  |  |  |  | x |  |

Funding agencies by country/region. Australia: Australian Government; National Health and Medical Research Council; Austria: Wissenschaftsfonds FWF (Fonds zur Förderung der wissenschaftlichen Forschung); Canada: Canadian Institutes of Health Research; Germany: Deutsche Forschungsgemeinschaft ; United Kingdom: Medical Research Council ; United States of America: National Institutes of Health ; Norway: The Research Council of Norway; Switzerland: Swiss National Science Foundation; Europe: Horizon2020

**B) Examples of criteria used by funding agencies to evaluate research proposals, by quality theme**

| Overarching quality themes | Examples (quotes) |
| --- | --- |
| Quality of researcher, applicant, and/or team | “*Qualifications of the applicant(s), including training, experience and independence (relative to career stage).*   - *Experience of the applicant(s) in the proposed area of research and with the proposed methodology.* - *Expertise of the applicant(s), as demonstrated by scientific productivity over the past five years (publications, books, grants held, etc.). Productivity should be considered in the context of the norms for the research area, applicant experience and total research funding of the applicant.* - *Ability to successfully and appropriately disseminate research findings, as demonstrated by knowledge translation activities (publications, conference presentations, briefings, media engagements, etc.).* - *Appropriateness of the team of applicants (if more than one applicant) to carry out the proposed research, in terms of complementarity of expertise and synergistic potential.” (****Canadian Institutes of Health Research)***   *“Applicants must have the professional competencies and qualifications required to complete the proposed action or work programme: it may be assessed on the basis of specific qualifications, professional experience and references in the field concerned”* ***(Horizon2020)*** |
| Impact and/or significance of research project | *“Assess the potential economic and social impact of the proposed research including:*   - *Identification of realistic improvements to human or population health* - *Contribution to relieving disease/disability burden and/or improving quality of life* - *Identification of potential impacts of research and plans to deliver these”* ***(Medical Research Council, UK)***   *“Does the project address an important problem or a critical barrier to progress in the field? If the aims of the project are achieved, how will scientific knowledge, technical capability, and/or clinical practice be improved? How will successful completion of the aims change the concepts, methods, technologies, treatments, services, or preventative interventions that drive this field? “* ***(National Institutes of Health, USA)*** |
| Quality of scientific approach and/or methods | *“This criterion gives an indication of the essential, fundamental aspects of the research project. The scientific merit of a project will be assessed in relation to the following points:*   - *Originality in the form of scientific innovation and/or the development of new knowledge.* - *Whether the research questions, hypotheses and objectives have been clearly and adequately specified.* - *The strength of the theoretical approach, operationalisation and use of scientific methods.* - *Documented knowledge about the research front.* - *The degree to which the scientific basis of the project is realistic.* - *The scientific scope in terms of a multi- and interdisciplinary approach, when relevant.”* ***(The Research Council of Norway)*** |
| Research infrastructure & financial capacity at applying site | *“Availability and accessibility of personnel, facilities and infrastructure required to conduct the research.*   - *Suitability of the environment to conduct the proposed research.* - *Suitability of the environment (milieu, project and mentors) for the training of personnel (if applicable).”* ***(Canadian Institutes of Health Research)***   *“Will the scientific environment in which the work will be done contribute to the probability of success? Are the institutional support, equipment and other physical resources available to the investigators adequate for the project proposed? Will the project benefit from unique features of the scientific environment, subject populations, or collaborative arrangements?”* ***(National Institutes of Health, USA)*** |
| Consideration of diversity & equal opportunities of applicants | *“Proposal reviews should not disadvantage applicants due to extra-scientific reasons, such as age, gender or disability. Consider the applicant’s scientific career development rather than his/her age. You may compensate for certain extra-scientific disadvantages; unavoidable delays in the applicant’s scientific career (for example childcare responsibilities causing longer periods of qualification, gaps in publications, or less time spent abroad) should be taken into consideration.”* ***(Deutsche Forschungsgemeinschaft, Germany)*** |
| Ethical considerations of research project | *“Does the project give rise to any ethical issues?*  ***(Wissenschaftsfonds FWF, Austria)*** |
| Originality/innovation of research project | *“Originality of the research question”* ***(Swiss National Science Foundation)*** |
| Feasibility of research project | *“(…) and feasibility of the proposal in terms of strengths and weaknesses”*  **(*Wissenschaftsfonds FWF, Austria)*** |
| Requested resources or funding adequate for proposed project | *“Justification of the proposed staff needs by the work programme*  *(…) Necessity and utilisation of the proposed instruments”* ***(Deutsche Forschungsgemeinschaft, Germany)*** |
| Quality of international / national cooperation arrangements | *For special programs: “Quality and add-on value of cooperation arrangements”* ***(Deutsche Forschungsgemeinschaft, Germany)***  *Additional criteria: “Quality of national and international cooperation”* ***(The Research Council of Norway)*** |
| Dissemination & communication of results | *“This criterion gives an indication of the quality of the dissemination and communication plans for the project. Dissemination and communication of results will be assessed in relation to the following points: • Plans for scholarly publication, dissemination and other communication activities. • Plans for popular science dissemination and communication activities vis-à-vis the general public as well as users of the project results, including planned use of channels and measures. • Plans for ensuring that important users (in industry, community life and public administration) are incorporated into/take part in dissemination activities for the project. When assessing dissemination and communication plans, importance should be attached to the level of detail provided and how realistic the plans are****.” (The Research Council of Norway)*** |
