# Supplementary material for: Towards the development of a comprehensive framework: Qualitative systematic survey of definitions of clinical research quality
Source: PLoS One. 2017 Jul 17;12(7):e0180635. doi: 10.1371/journal.pone.0180635 (PMC5513422; doi:10.1371/journal.pone.0180635)
Supplement: S1 Text — (DOCX) [file pone.0180635.s006.docx]

**S1 Text. Literature search strategy**

1 Quality Control/

2 total quality management/

3 Quality Improvement/

4 Quality Indicators, Health Care/

5 exp *Reproducibility of Results/

6 good clinical practic*.mp.

7 or/1-6

8 biomedical research/ or clinical nursing research/

9 (biomedical and research).ti,ab.

10 (clinical adj2 research).ti,ab.

11 Randomized Controlled Trial* as Topic.mp. or Randomized Controlled Trials as Topic/

12 clinical trial* as topic.mp. or Clinical Trials as Topic/

13 Research/st [Standards]

14 or/8-13

15 7 and 14

16 ((quality or valid* or data integrity) adj4 (clinical stud* or clinical data or clinical trial* or randomized trial* or randomised trial* or control* trial*)).ti,ab.

17 ((quality or valid* or data integrity) adj4 (clinical or medical or human* or patient*) adj2 research*).ti,ab.

18 16 or 17

19 15 or 18

20 or/1-6

21 14 and 20

22 18 or 21

23 (approach or assessment* or assessing or assurance or checklist* or check list*).tw.

24 (code of conduct or concepts or concept or clinimetric* or definition* or evaluation*).tw.

25 (framework* or guideline* or guidance or instrument or instruments or indicators or indicator).tw.

26 (predictor* or measurement* or measures or measure or process or rating*).tw.

27 (scale? or score? or standard? or norm? or system? or tool? or dimension? or item?).tw.

28 (factor? or criteria or principle? or grading or grade or attributes or metrics or monitor).tw.

29 (recommendation? or priority or priorities or construct? or determinant? or project?).tw.

30 (report? or point? or categories or category or summaries or summary or ranking?).tw.

31 (statistic? or term? or feature? or characteristic? or profile? or pattern? or rule? or idea).tw.

32 (theory or paradigm? or consensus or statement or promotion or promoting or test?).tw.

33 (improvement* or improving or increasing).tw.

34 or/23-33

35 22 and 34
