# Supplementary material for: Towards the development of a comprehensive framework: Qualitative systematic survey of definitions of clinical research quality
Source: PLoS One. 2017 Jul 17;12(7):e0180635. doi: 10.1371/journal.pone.0180635 (PMC5513422; doi:10.1371/journal.pone.0180635)
Supplement: S2 Text — (DOCX) [file pone.0180635.s007.docx]

**S2 Text. References of eligible articles**

[1] Berger VW, Alperson SY. A general framework for the evaluation of clinical trial quality. Rev Recent Clin Trials. 2009;4:79-88.

[2] Berk PD, Sacks HS. Assessing the quality of randomized controlled trials: quality of design is not the only relevant variable. Hepatology. 1999;30:1332-4.

[3] Verhagen AP, de Vet HC, de Bie RA, Boers M, van den Brandt PA. The art of quality assessment of RCTs included in systematic reviews. J Clin Epidemiol. 2001;54:651-4.

[4] Krestin GP. Evaluating the Quality of Radiology Research: What Are the Rules of the Game? Radiology. 2008;249:418-24.

[5] Dechartres A, Charles P, Hopewell S, Ravaud P, Altman DG. Reviews assessing the quality or the reporting of randomized controlled trials are increasing over time but raised questions about how quality is assessed. J Clin Epidemiol. 2011;64:136-44.

[6] Moher D, Jadad AR, Tugwell P. Assessing the quality of randomized controlled trials. Current issues and future directions. Int J Technol Assess Health Care. 1996;12:195-208.

[7] Clark GT, Mulligan R. Fifteen common mistakes encountered in clinical research. J Prosthodont Res. 2011;55:1-6.

[8] Kleppinger CF, Ball LK. Building quality in clinical trials with use of a quality systems approach. Clin Infect Dis. 2010;51 Suppl 1:S111-6.

[9] Rosenblum D, Alving B. The role of the clinical and translational science awards program in improving the quality and efficiency of clinical research. Chest. 2011;140:764-7.

[10] Weihrauch TR, Baumbauer E. [Drug research in Germany--problems and chances--from the viewpoint of the drug industry]. Arzneimittelforschung. 1998;48:1047-50.

[11] Beger HG, Schwarz A. Clinical research in surgery: questions but few answers. Langenbecks Arch Surg. 1998;383:300-5.

[12] Prescott RJ, Counsell CE, Gillespie WJ, Grant AM, Russell IT, Kiauka S, et al. Factors that limit the quality, number and progress of randomised controlled trials. Health Technol Assess. 1999;3:1-143.

[13] Njie VP, Thomas AC. Quality issues in clinical research and the implications on health policy (QICRHP). J Prof Nurs. 2001;17:233-42.

[14] Holland WW, Mosbech J. International quality control of national scientific standards. J Intern Med. 1993;234:1-2.

[15] Knatterud GL, Rockhold FW, George SL, Barton FB, Davis CE, Fairweather WR, et al. Guidelines for quality assurance in multicenter trials: a position paper. Control Clin Trials. 1998;19:477-93.

[16] Strause LG, Vogel JR. The clinical research triad: how can we ensure quality in out-sourced clinical trials? Qual Manag Health Care. 1999;7:23-9.

[17] American Society of Clinical O. American Society of Clinical Oncology policy statement: oversight of clinical research. J Clin Oncol. 2003;21:2377-86.

[18] Ottevanger PB, Therasse P, van de Velde C, Bernier J, van Krieken H, Grol R, et al. Quality assurance in clinical trials. Crit Rev Oncol Hematol. 2003;47:213-35.

[19] Franck LS, Pendleton E, Pittam B, Preece M, Aynsley-Green A. Quality assurance for clinical research: challenges in implementing research governance in UK hospitals. Int J Health Care Qual Assur Inc Leadersh Health Serv. 2004;17:239-47.

[20] Warden D, Rush AJ, Trivedi M, Ritz L, Stegman D, Wisniewski SR. Quality improvement methods as applied to a multicenter effectiveness trial--STAR D. Contemp Clin Trials. 2005;26:95-112.

[21] Switula D. The concept of quality in clinical research. Sci Eng Ethics. 2006;12:147-56.

[22] Zon R, Meropol NJ, Catalano RB, Schilsky RL. American Society of Clinical Oncology Statement on minimum standards and exemplary attributes of clinical trial sites. J Clin Oncol. 2008;26:2562-7.

[23] Bhatt A. Quality of clinical trials: A moving target. Perspect Clin Res. 2011;2:124-8.

[24] Hanna M, Minga A, Fao P, Borand L, Diouf A, Mben JM, et al. Development of a checklist of quality indicators for clinical trials in resource-limited countries: the French National Agency for Research on AIDS and Viral Hepatitis (ANRS) experience. Clin. 2013;10:300-18.

[25] Powe NR, Kinnison ML, Steinberg EP. Quality assessment of randomized controlled trials of contrast media. Radiology. 1989;170:377-80.

[26] Newcastle Ottawa Scale.

[27] Chalmers TC, Smith H, Jr., Blackburn B, Silverman B, Schroeder B, Reitman D, et al. A method for assessing the quality of a randomized control trial. Control Clin Trials. 1981;2:31-49.

[28] Cho MK, Bero LA. Instruments for assessing the quality of drug studies published in the medical literature. Jama. 1994;272:101-4.

[29] Jefferson T, Wager E, Davidoff F. Measuring the quality of editorial peer review. Jama. 2002;287:2786-90.

[30] Jadad AR, Moore RA, Carroll D, Jenkinson C, Reynolds DJ, Gavaghan DJ, et al. Assessing the quality of reports of randomized clinical trials: is blinding necessary? Control Clin Trials. 1996;17:1-12.

[31] Manchikanti L, Hirsch JA, Cohen SP, Heavner JE, Falco FJ, Diwan S, et al. Assessment of methodologic quality of randomized trials of interventional techniques: development of an interventional pain management specific instrument. Pain physician. 2014;17:E263-90.

[32] Kocsis JH, Gerber AJ, Milrod B, Roose SP, Barber J, Thase ME, et al. A new scale for assessing the quality of randomized clinical trials of psychotherapy. Compr Psychiatry. 2010;51:319-24.

[33] Nass SJ, Balogh E, Mendelsohn J. A National Cancer Clinical Trials Network: recommendations from the Institute of Medicine. Am J Ther. 2011;18:382-91.

[34] Arrive L, Renard R, Carrat F, Belkacem A, Dahan H, Le Hir P, et al. A scale of methodological quality for clinical studies of radiologic examinations. Radiology. 2000;217:69-74.

[35] Balk EM, Bonis PA, Moskowitz H, Schmid CH, Ioannidis JP, Wang C, et al. Correlation of quality measures with estimates of treatment effect in meta-analyses of randomized controlled trials. Jama. 2002;287:2973-82.

[36] Barske HL, Baumhauer J. Quality of research and level of evidence in foot and ankle publications. Foot Ankle Int. 2012;33:1-6.

[37] Birch S. Clinical research on acupuncture. Part 2. Controlled clinical trials, an overview of their methods. J Altern Complement Med. 2004;10:481-98.

[38] Bizzini M, Childs JD, Piva SR, Delitto A. Systematic review of the quality of randomized controlled trials for patellofemoral pain syndrome. J Orthop Sports Phys Ther. 2003;33:4-20.

[39] Bornhoft G, Maxion-Bergemann S, Wolf U, Kienle GS, Michalsen A, Vollmar HC, et al. Checklist for the qualitative evaluation of clinical studies with particular focus on external validity and model validity. BMC Med Res Methodol. 2006;6:56.

[40] Coleman BD, Khan KM, Maffulli N, Cook JL, Wark JD. Studies of surgical outcome after patellar tendinopathy: clinical significance of methodological deficiencies and guidelines for future studies. Victorian Institute of Sport Tendon Study Group. Scand J Med Sci Sports. 2000;10:2-11.

[41] Tate RL, McDonald S, Perdices M, Togher L, Schultz R, Savage S. Rating the methodological quality of single-subject designs and n-of-1 trials: introducing the Single-Case Experimental Design (SCED) Scale. Neuropsychol. 2008;18:385-401.

[42] Yates SL, Morley S, Eccleston C, de CWAC. A scale for rating the quality of psychological trials for pain. Pain. 2005;117:314-25.

[43] Scholler K, Licht S, Tonn JC, Uhl E. Randomized controlled trials in neurosurgery--how good are we? Acta Neurochir (Wien). 2009;151:519-27; discussion 27.

[44] Sindhu F, Carpenter L, Seers K. Development of a tool to rate the quality assessment of randomized controlled trials using a Delphi technique. J Adv Nurs. 1997;25:1262-8.

[45] Manchikanti L, Hirsch JA, Heavner JE, Cohen SP, Benyamin RM, Sehgal N, et al. Development of an interventional pain management specific instrument for methodologic quality assessment of nonrandomized studies of interventional techniques. Pain physician. 2014;17:E291-317.

[46] Balshem H, Helfand M, Schunemann HJ, Oxman AD, Kunz R, Brozek J, et al. GRADE guidelines: 3. Rating the quality of evidence. J Clin Epidemiol. 2011;64:401-6.

[47] Downs SH, Black N. The feasibility of creating a checklist for the assessment of the methodological quality both of randomised and non-randomised studies of health care interventions. J Epidemiol Community Health. 1998;52:377-84
